# Progression of diabetic retinopathy in a longitudinal real-world study of patients in primary care

**DOI:** 10.1186/s12886-025-04307-1

**Published:** 2025-10-06

**Authors:** Geeta Lalwani, Charles C. Wykoff, Jayla Briggs, Chin-Yu Lin, Verena Steffen, Zdenka Haskova

**Affiliations:** 1 Rocky Mountain Retina Associates, 1440 28th Street, Suite 3, Boulder, CO 80303 USA; 2 Retina Consultants of Texas, Retina Consultants of America, Blanton Eye Institute Houston Methodist Hospital, Houston, TX USA; 3 Genentech, Inc., South San Francisco, CA USA

**Keywords:** Diabetes mellitus, Diabetic retinopathy, Clinically significant macular edema, Non-proliferative diabetic retinopathy, Proliferative diabetic retinopathy

## Abstract

**Background:**

The aim of this study was to assess the impact of diabetic retinopathy (DR) severity on the risk of DR progression to proliferative DR (PDR) or clinically significant macular edema (CSME) in patients with diabetes mellitus (DM).

**Methods:**

This is a prospective, longitudinal, non-interventional, and observational cohort study of patients with DM in the United States based on a database of 7-field color fundus photograph images from primary care visits. Study participants were adults aged ≥ 18 years who underwent DR screening in either eye between January 1999 and December 2016. DR severity was graded according to the Early Treatment Diabetic Retinopathy Study-Diabetic Retinopathy Severity Scale (ETDRS-DRSS) on 7-field color fundus photographs. The main outcomes were ≥ 2-step DR worsening and development of CSME, PDR, or CSME and PDR together.

**Results:**

For all 41,977 eyes evaluated, the proportion of eyes with ≥ 2-step DR worsening was 2.7% at year 2 and 9.6% at year 5. Rate of ≥ 2-step DR worsening was greatest among eyes with moderate-to-severe NPDR with baseline DRSS 43–53 (36.5% at year 5). Analysis of PDR and CSME outcomes showed the presence of 3 distinct clinical phenotypes: 1 subset progressed to CSME (1.24% at year 5), another to PDR (0.49% at year 5), and only a small subset progressed to both vision-threatening forms of DR (0.10% at year 5). The clinical phenotype did not appear to be dependent on baseline DRSS.

**Conclusions:**

Overall, the risk of progression to vision-threatening forms of DR was more pronounced in eyes with moderate-to-severe non-proliferative DR at baseline. In addition, we found that distinct DR clinical subtypes progressing to either PDR and/or CSME over a 5-year period are not driven by baseline DR severity, suggesting other factors may contribute.

**Supplementary Information:**

The online version contains supplementary material available at 10.1186/s12886-025-04307-1.

## Background

Diabetic retinopathy (DR), one of the most common complications of diabetes mellites (DM), is a leading cause of preventable blindness and vision loss in working-aged adults [1–4]. DR progresses insidiously, from mild, moderate, then severe non-proliferative DR (NPDR) to vision-threatening proliferative DR (PDR), without easily detectable retinal abnormalities and no or minimal symptoms through all stages of NPDR [5–7]. Although factors associated with DR severity progression are known, including duration of DM, age at diagnosis, HbA1c levels, blood pressure, insulin use, and presence of proteinuria [8], PDR is estimated to occur in about 8% patients with DM [4, 9]. Central vision can quickly deteriorate and patients can develop diabetic macular edema (DME) [10] and clinically significant macular edema (CSME) at any stage of DR.

To assess severity and change in severity of DR, the Early Treatment Diabetic Retinopathy Study-Diabetic Retinopathy Severity Scale (ETDRS-DRSS; scale from 1 to 17+) was developed [11]. Although not routinely used in clinical practice in its detailed format, ETDRS-DRSS was validated as a predictive assessment of DR progression and remains the gold standard for endpoint evaluation in clinical trials [12]. Greater than 2-step ETDRS-DRSS progression (i.e., an increase in DRSS scale of ≥ 2 steps) has been determined to be a reliable clinically meaningful measure, especially in clinical trials settings [10, 13]. For example, studies have found that an ≥ 1-step of progression over a 4-year period was strongly associated with the development of PDR or CSME over the next 6 years [14].

Using a large historical database of color fundus photographs from patients with DM, the aim of our study was to explore the natural progression of DR to vision-threatening PDR and/or CSME in the eyes of patients screened at primary care centers across the United States using 2-step ETDRS-DRSS progression. This method was selected for assessment and for classification of CSME (defined by where retinal thickening and/or hard exudates adjacent to thickening occur at or within 500 μm of the fovea) [15] because some of the database precedes the introduction of ocular coherence tomography, which is currently routinely used for assessment of DR progression. Importantly, although clinical trials have reported that the risk of developing PDR or CSME increases with DR severity level at baseline [16, 17], this has not been assessed in a large population of patients with DM from a real-world setting. We report changes in ETDRS-DRSS and progression to vision-threatening forms of DR (PDR or CSME) in the overall population and among eyes with moderate-to-severe NPDR at baseline, as these patients are at the greatest risk of DR progression.

## Methods

### Study design

This study is a prospective, longitudinal, non-interventional, and observational cohort study of patients with DM in the United States based on a database of 7-field color fundus photograph images. These de-identified data were purchased by Roche/Genentech from Inoveon (Inoveon Corporation, Oklahoma City, OK, USA), a retinal imaging company that was previously used for monitoring DR in the DM primary care setting. Given this study utilizes a database of images, information on management of patients before and after imaging is not available. The study was approved by the University of Oklahoma Health Sciences Center Institutional Review Board (#INV-001) and participants provided written informed consent prior to capture of photographs.

Diabetic retinopathy severity and CSME were assessed from 7-field color fundus photographs by professional graders at a centralized reading center at Vanderbilt University Medical Center, Nashville, TN, USA; images were double graded by readers masked to the other readers’ results, with independent third reader adjudication. DR severity was classified according to the ETDRS-DRSS, which categorizes DR into multiple levels, including absent (level 10), mild microaneurysm only (level 20), mild NPDR (level 35), moderate NPDR (level 43), moderately severe NPDR (level 47), and severe NPDR (level 53); and mild PDR (level 61), moderate PDR (level 65), high-risk PDR (level 71/75), and advanced PDR (level 81/85) [18].

Due to the historical nature of this database, optical coherence tomography images are not available and patient data are limited to age, sex, race, and diabetes type.

### Analysis population

Patients with DM ≥ 18 years of age were screened for DR between January 1999 and December 2016 and captured in the Inoveon database. Only patients with images that were associated with ≥ 2 valid ETDRS-DRSS assessments in either eye were included in this analysis.

### Outcomes

Clinically relevant DR worsening in an eye was defined by an ≥ 2-step increase on the ETDRS-DRSS compared with the initial assessment. The development of CSME (using ETDRS criteria) and/or PDR was analyzed among eyes with no CSME and no PDR at baseline. In addition, in a subanalysis, the impact of race and DM type on time to first ≥ 2-step DR worsening was examined.

Definitions of the main outcome measures are provided in Supplementary Table 1.

The number of eyes at risk was determined as follows. Patients who had color fundus photographs evaluable for the DRSS score in ≥ 2 visits in either eye were included. The “Number of eyes at risk of developing DR at year 1” refers to the number of eyes from patients who had ≥ 1 year of follow-up data available. Similar definitions were applied for “Number of eyes at risk” for years 2, 3, 4, and 5, including only eyes from patients with follow-up data available for those respective time points.

### Statistical analysis

Distributions of time to first ≥ 2-step DR worsening and development of CSME, PDR, or CSME and PDR were estimated using the Kaplan-Meier method. Time to first diagnosis of ≥ 2-step DR worsening was summarized by the initial DRSS score.

## Results

### Patient characteristics

**Fig. 1:**
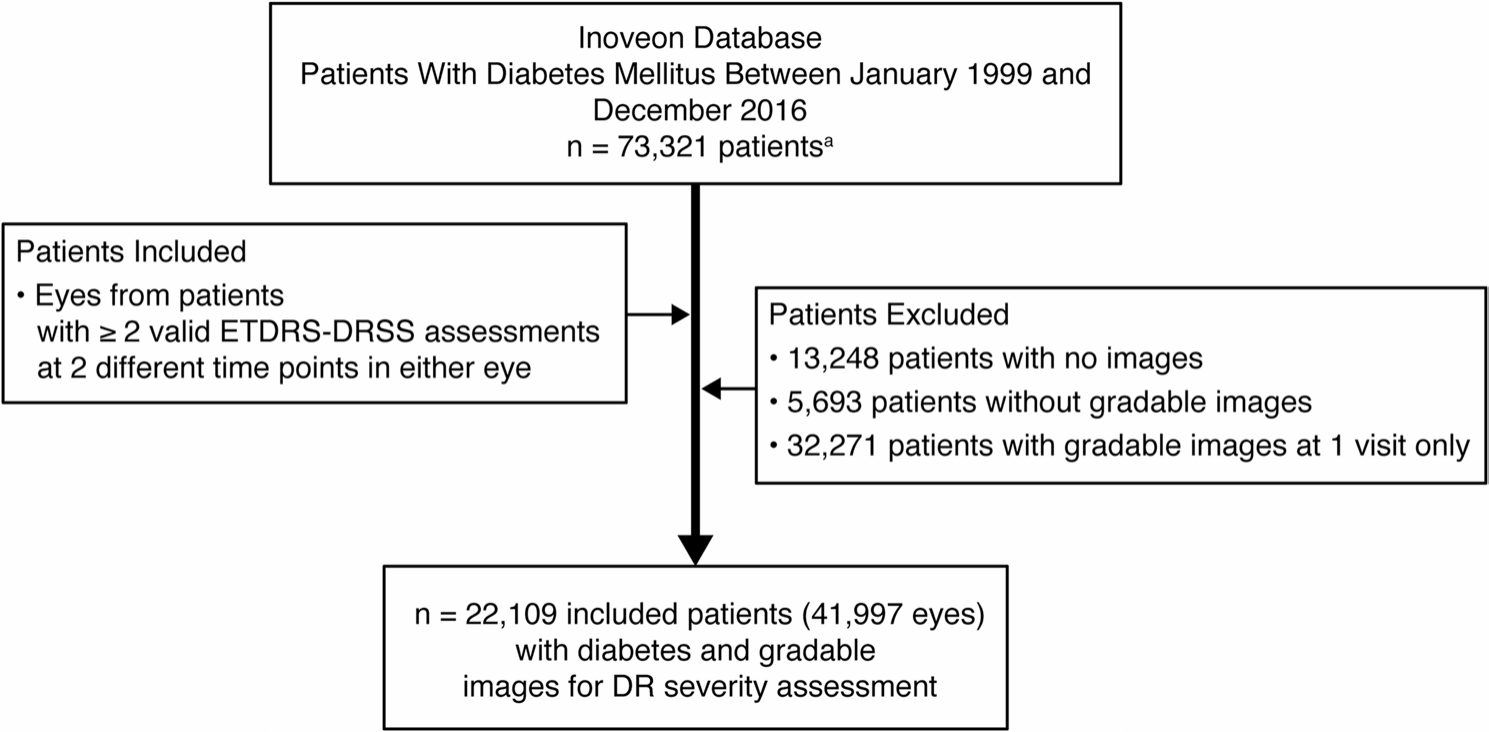
Patient selection flowchart. ^a^Excludes test cases. DR: diabetic retinopathy, ETDRS-DRSS: Early Treatment Diabetic Retinopathy Study-Diabetic Retinopathy Severity Scale

Between January 1999 and December 2016, the Inoveon database collected images and data from 73,321 unique patients with DM. A total of 51,212 patients were excluded due to no images, or no gradable images at ≥ 2 visits; the remaining 22,109 patients (41,997 eyes) constituted the study population (Fig. 1). Baseline patient demographics are presented in Table 1; median age was 61 years and most patients with known status were male (83.0%). Only a subset of patients (*n* = 4,085) reported their race or ethnicity; most patients who provided this information were White (48.0%) or Native American (41.0%). Only a subset of patients (*n* = 11,334) reported diabetes type; most patients who provided this information had type 2 diabetes (94.2%). Most patients resided in Oklahoma (44.3%) or Florida (31.4%).

**Table 1:** Baseline demographics of the patients included in the study

Characteristic	Total study population
Summary by patient	
Number of patients	22,109
Median age (range), years	61 (6‒85)
Sex, *n* (%)^a^	
Male	14,021 (83.0)
Female	2,876 (17.0)
Unknown	5,212
Race, *n* (%)^a^	
White	1,948 (48.0)
Native American	1,662 (41.0)
African American/Black	369 (9.1)
Hispanic	50 (1.2)
Other	29 (0.7)
Unknown	18,051
Diabetes mellitus type, *n* (%)^a^	
Type 1	653 (5.8)
Type 2	10,676 (94.2)
Gestational	5 (< 0.1)
Unknown	10,775
Summary by eye	
Number of eyes	41,977
Baseline DRSS level, *n* (%)	
10	32,219 (76.7)
14	1,234 (2.9)
15	1,687 (4.0)
20	2,420 (5.8)
35	3,166 (7.5)
43	639 (1.5)
47	266 (0.6)
53	104 (0.3)
61	138 (0.3)
65	87 (0.2)
71	31 (0.1)
75	5 (0.0)
81	1 (0.0)

^a^Proportions calculated for patients with known data for each variable

*DRSS *Diabetic Retinopathy Severity Scale

### Progression of DR by ETDRS-DRSS

**Fig. 2:**
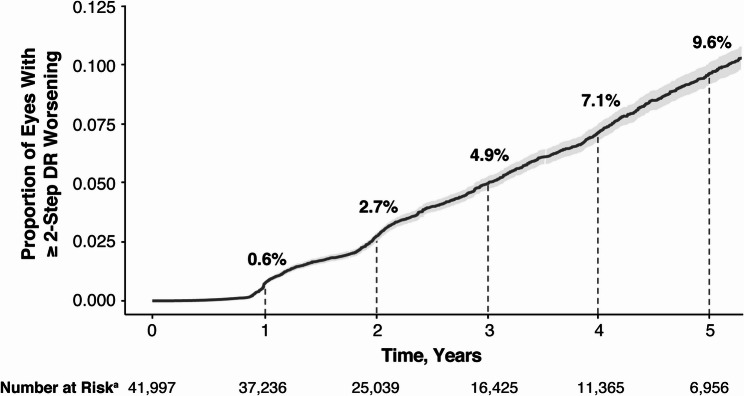
≥ 2-step DR worsening in all eyes. Data from 41,997 eyes from 22,109 patients ^a^Number of eyes at risk of developing DR at each year. Shaded region indicates 95% CI. CI: confidence interval, DR: diabetic retinopathy, ETDRS-DRSS: Early Treatment Diabetic Retinopathy Study-Diabetic Retinopathy Severity Scale

**Fig. 3:**
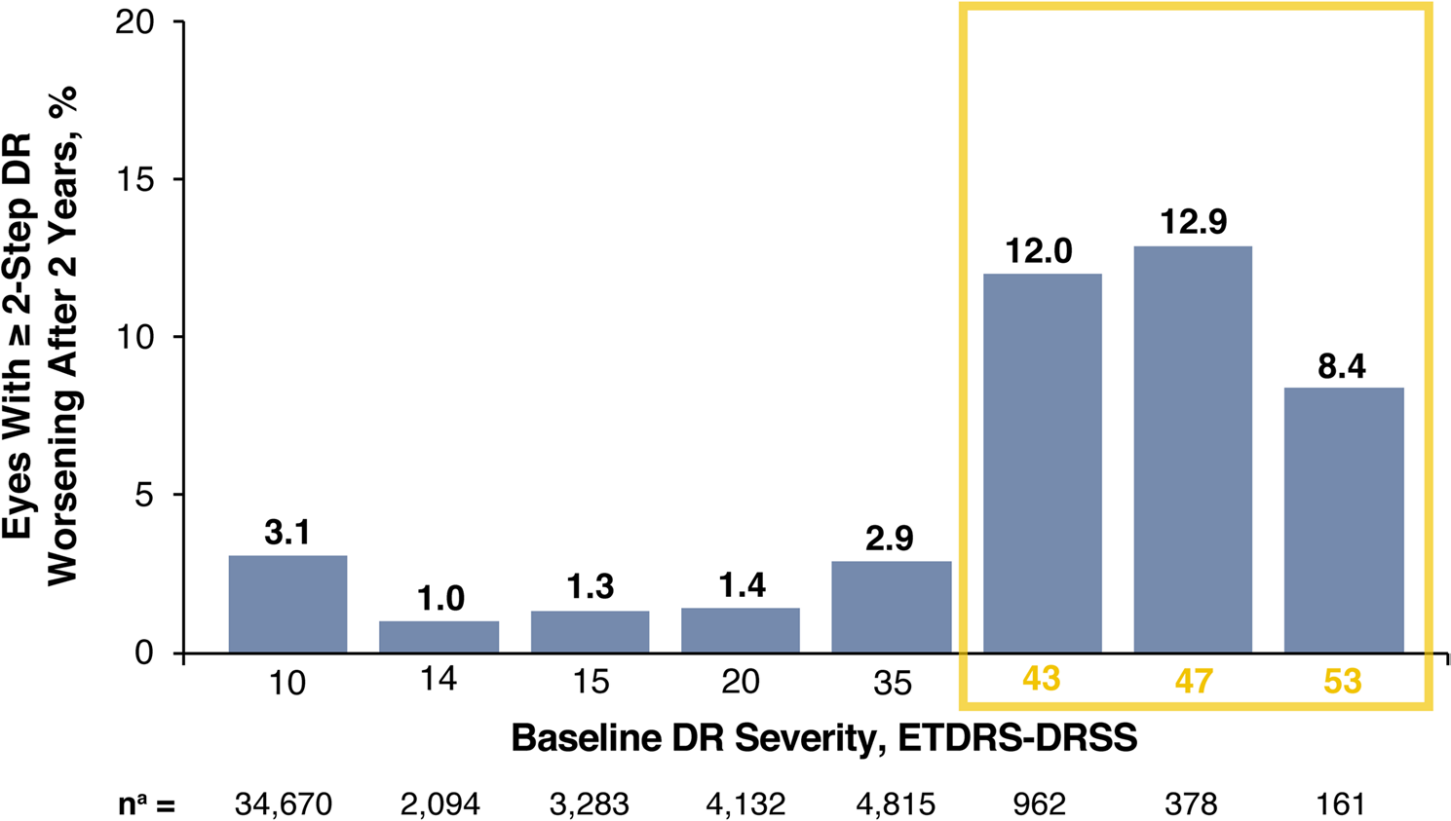
Percentage of eyes with ≥ 2-step DR worsening at year 2 based on baseline DR severity. Yellow box indicates the moderate-to-severe NPDR eyes. ^a^Each eye could be included in multiple baseline severity subtypes. DR: diabetic retinopathy, ETDRS-DRSS: Early Treatment Diabetic Retinopathy Study-Diabetic Retinopathy Severity Scale

**Fig. 4:**
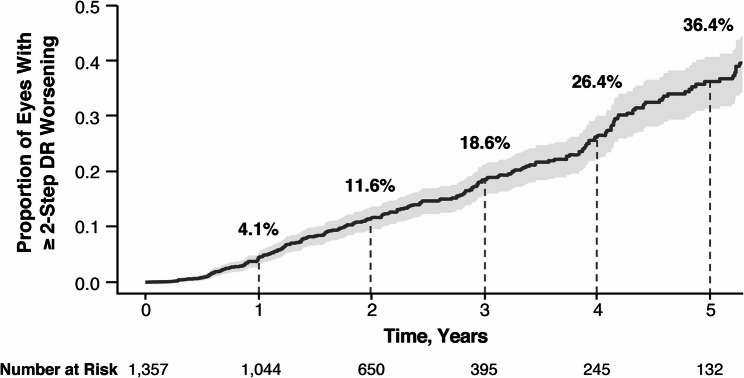
≥ 2-step DR worsening in eyes with ETDRS-DRSS 43‒53 at baseline. Data from 999 patients and 1,357 eyes. Number of eyes at risk of developing DR at each year. Shaded region indicates 95% CI. CI: confidence interval, DR: diabetic retinopathy, ETDRS-DRSS: Early Treatment Diabetic Retinopathy Study-Diabetic Retinopathy Severity Scale

For all eyes evaluated, the proportion of eyes with ≥ 2-step DR worsening was 2.7% at year 2, 7.1% at year 4, and 9.6% at year 5 (Fig. 2). As expected, the percentage of eyes with ≥ 2-step DR worsening after year 2 was greatest among eyes with moderate-to-severe NPDR (level 43–53) at baseline (Fig. 3). For the pooled moderate-to-severe NPDR (level 43–53) eyes at baseline, the proportion of eyes with ≥ 2-step worsening was 11.6% at year 2, 26.4% at year 4, and 36.4% at year 5 in this subset (Fig. 4). Similar findings were reported among eyes with moderately severe to severe NPDR (level 47–53) at baseline (Supplementary Fig. 2).

### Progression of DR by PDR and/or CSME

**Fig. 5:**
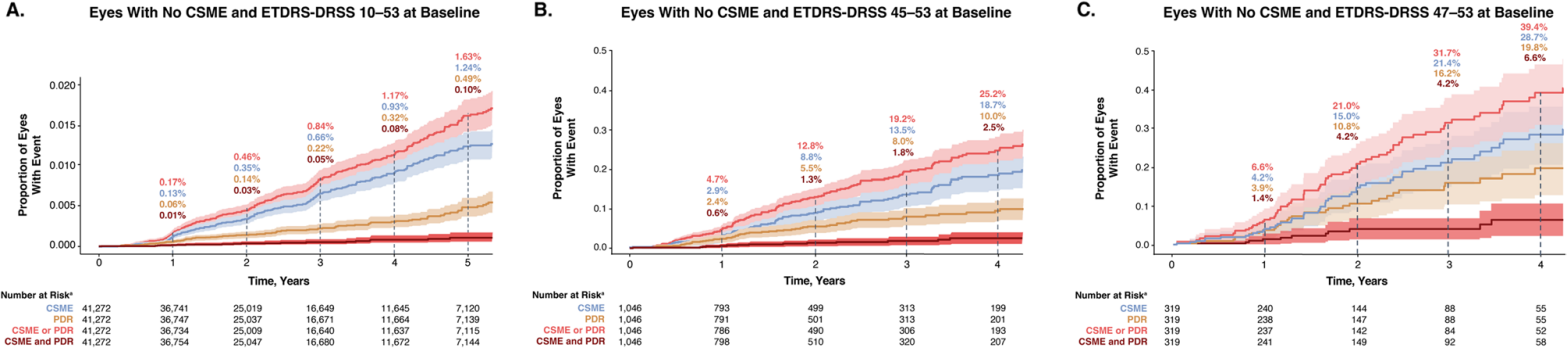
Development of CSME, PDR, or CSME and PDR over time in **a** Eyes with no CSME and ETDRS-DRSS 10–53 at baseline. Data from 21,757 patients and 41,272 eyes, **b** Eyes with no CSME and ETDRS-DRSS 43–53 at baseline. Data from 810 patients and 1,046 eyes, and **c** Eyes with no CSME and ETDRS-DRSS 47–53 at baseline. Data from 265 patients and 310 eyes. ^a^Number of eyes at risk of developing CSME, PDR, or either CSME or PDR, at each year. Shaded regions indicate 95% CI. CI: confidence interval, CSME: clinically significant macular edema, ETDRS-DRSS: Early Treatment Diabetic Retinopathy Study-Diabetic Retinopathy Severity Scale, PDR: proliferative diabetic retinopathy

Analysis of PDR and/or CSME development over time in all available eyes with no CSME and no PDR (ETDRS-DRSS 10–53) at baseline suggested the existence of 3 distinct clinical phenotypes: 1 progressed to CSME (1.24% at year 5), another progressed to PDR (0.49% at year 5), and only a small subset experienced progression to both vision-threatening forms of DR (0.10% at year 5; Fig. 5a). Existence of these 3 distinct clinical DR phenotypes did not appear to be driven by baseline DR severity: similar trends of distinct clinical progression subtypes were observed in eyes with no CSME and moderate-to-severe NPDR (ETDRS-DRSS 45–53) at baseline (Fig. 5b), and no CSME and moderately severe to severe NPDR (ETDRS-DRSS 47–53) at baseline (Fig. 5c). Patients with moderate-to-severe NPDR at baseline experienced higher rates of progression to PDR or CSME compared with the overall population with DR.

### Subanalysis by race

**Fig. 6:**
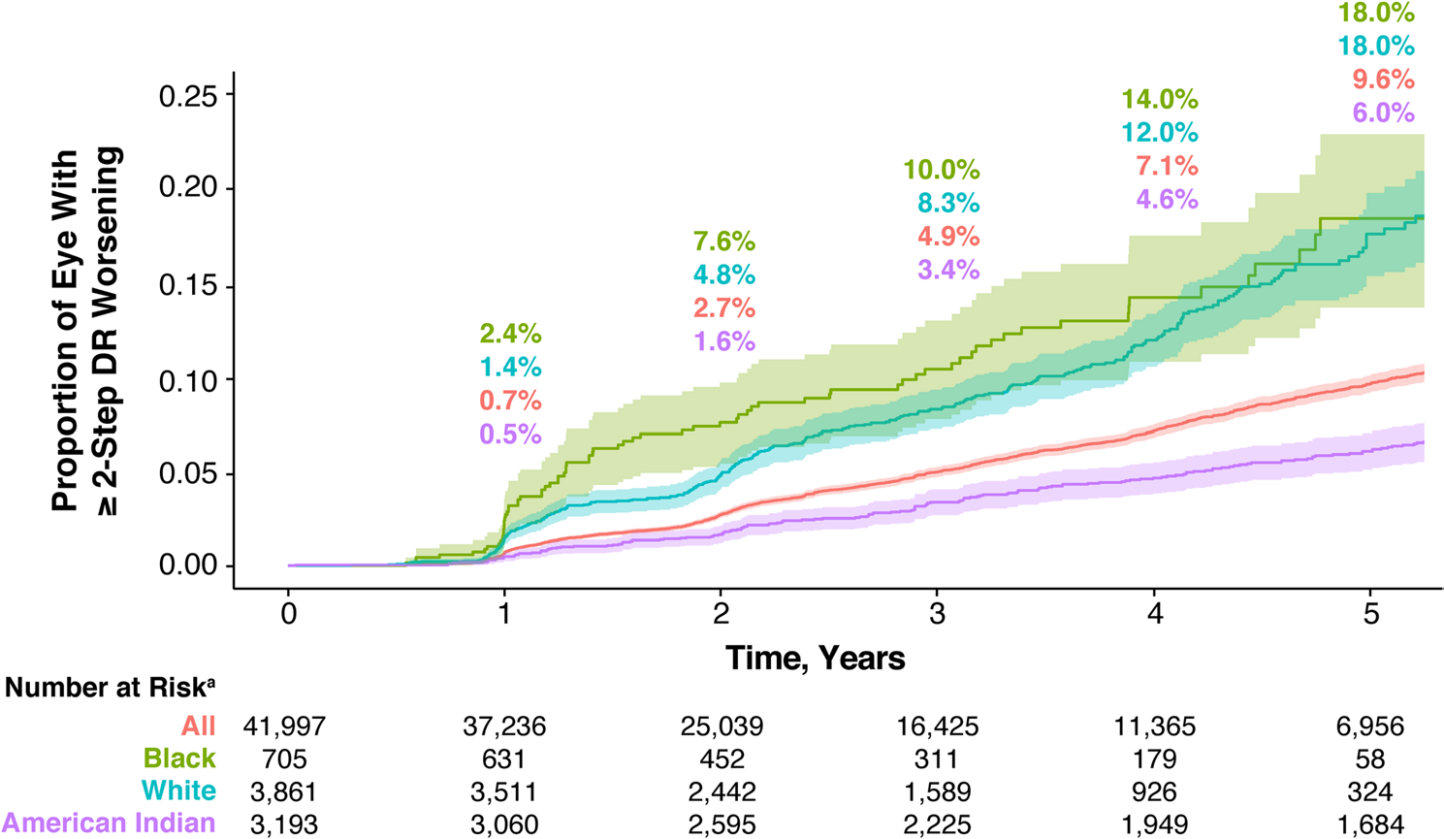
≥ 2-step DR worsening by race for all eyes. ^a^Number of eyes at risk for the first ≥ 2-step DR worsening from baseline at each year. Shaded regions indicate 95% CI. CI: confidence interval, DR: diabetic retinopathy

In the subanalysis of all eyes categorized by race, the rate of ≥ 2-step worsening was higher in Black and White patients compared with American Indian (now referred to as Native American) patients. By year 2, the proportion of eyes with ≥ 2-step worsening was 7.6% in Black, 4.8% in White, and 1.6% in Native American patients (Fig. 6).

### Subanalysis by DM type

**Fig. 7:**
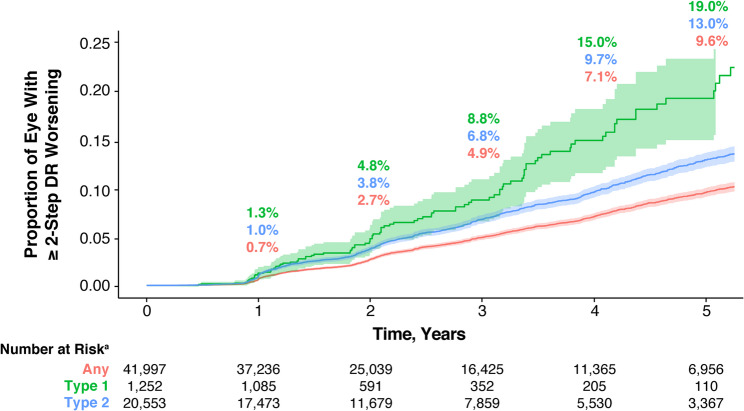
≥ 2-step DR worsening by diabetes mellitus type for all eyes. ^a^Number of eyes at risk for ≥ 2-step DR worsening from baseline at each year. Shaded regions indicate 95% CI. CI: confidence interval, DR: diabetic retinopathy

In the subanalysis of all eyes categorized by DM type, the estimated proportion of DR worsening by ≥ 2 steps was higher with type 1 DM compared with type 2 DM (Fig. 7).

## Discussion

Using an atypical approach of analyzing a historical database of 7-field color fundus photographs, we completed a comprehensive evaluation of DR progression in patients with DM screened at primary care centers (without retinal specialist consult) across the United States. For patients with DR, our study highlights the likelihood of progression to vision-threatening phenotypes. Overall, approximately 10% of all eyes in our analysis experienced an ≥ 2-step DR worsening by year 5. The risk of progression to vision-threatening forms of DR was more pronounced in eyes with moderate-to-severe NPDR at baseline, which is in line with previous studies [19]. Our key observation was the description of distinct clinical DR subtypes that progressed to either PDR or CSME, plus a relatively smaller subtype that progressed to both CSME and PDR. This finding appeared to be independent of baseline DR severity, although it is in line with findings from randomized clinical trials (although direct comparison to trials is not possible due to sample size and patient populations). For example, about 50% of eyes in the sham arm of the PANORAMA trial developed both a vision-threatening complication and/or center-involved DME (CI-DME) at week 100 [20], and 43.5% of patients in the sham arm of the Diabetic Retinopathy Clinical Research (DRCR) Network Protocol W study developed CI-DME by year 2 [21], noting the trials only recruited patients with moderately severe to severe NPDR and patients were assessed by retina specialists. Given early detection of vision-threatening DR through screening and retinal assessments can help in the prevention of vision loss resulting from DR [22, 23], insights from our study could help inform physicians and potentially improve patient care.

Our study also found a higher rate of progression to DR in patients with type 1 DM compared with type 2 DM. This is similar to previous studies such as Lee et al. [24], who reported a higher prevalence of any DR (77.3% vs. 25.2%) and PDR (32.4% vs. 3.0%) in patients with type 1 DM versus type 2 DM, and a higher prevalence of DME in patients with type 1 DM (4.2–7.9%) versus type 2 DM (1.4–12.8%). Even though comparisons between populations of type 1 and type 2 DM are limited by a higher proportion of type 2 DM (> 90% of all patients with DM in our study), this aligns with the real-world prevalence of these subtypes [25, 26] and highlights to physicians that patients with type 1 DM may require additional vision management, as they may be more at risk for vision-threatening DR.

The limitations of our study are primarily related to the nature of the database. The progression rate of NPDR to PDR and/or CSME may be underestimated because the patients did not return at regularly scheduled follow-up visits, and the analysis population included patients with ≥ 2 valid ETDRS-DRSS assessments in a primary care setting, thus excluding patients who transitioned to specialty care. Given the historical nature of the database and collection of data at primary care settings, the database does not include modern imaging modalities such as optical coherence tomography or optical coherence tomography angiography, which could have provided more detailed insights into structural and vascular changes in DR. In addition, clinical characteristics beyond those reported in this report, e.g., control of DM, duration of DM, or prior treatments/procedures for eye conditions, were not available in the database; thus, we were unable to assess DR in the context of other clinical outcomes. Interpretation of our analysis may be limited by the older data collection period (1999–2016) and the incomplete reporting of patient demographics, especially race, suggesting a potential selection bias. Nevertheless, we found a higher rate of ≥ 2-step DR worsening in the eyes of Black and White patients compared with the eyes of Native American patients, highlighting a racial disparity that warrants further investigation. Despite these limitations, the strength of our study is the large volume of high-quality color fundus photographs that were collected over a > 5-year period from a large real-world population of patients with DM in the United States. Although other studies have utilized large databases to assess DR [27, 28], to our knowledge, our study represents a novel attempt to conduct a large-scale imaging assessment in a primary care setting.

## Conclusions

In conclusion, our study of patients with DR within routine primary care clinical practice confirms that eyes with moderate-to-severe NPDR at baseline have the greatest risk of DR progression to both vision-threatening forms of PDR and CSME over a 5-year period. Our findings from this large real-world population of patients with DM screened in the United States go beyond previous observations from smaller populations in clinical trials. Our findings suggest that distinct DR clinical phenotypes are not solely driven by baseline DR severity, because DR phenotype remained consistent regardless of the baseline DRSS. Although guidelines exist to help prevent, delay, and manage DR – with initial screening within 5 years after onset of type 1 DM or at diagnosis for adults with type 2 DM, followed by at least annual screening if any level of DR is present [29] – physicians should be aware that DR may progress in some patients. Our real-world findings may help educate physicians about DR progression patterns and highlight the importance of early screening, which could facilitate timely diagnosis and physician implementation of strategies to delay progression, thus improving quality of life for patients. Future studies to identify potential factors that may contribute to the distinct clinical phenotypes in patients with progressing DR may help clinicians effectively manage the growing population of patients affected by DR.

## Supplementary Information

Supplementary Material 1.

## Data Availability

Due to the sensitivity and privacy around the Inoveon Health Database, raw data must remain confidential and cannot be shared. Aggregated data are available upon reasonable request to Genentech, Inc. (haskova.zdenka@gene.com).

## Abbreviations

CI-DME: Center-involved diabetic macular edema
CSME: Clinically significant macular edema
DM: Diabetes mellitus
DME: Diabetic macular edema
DR: Diabetic retinopathy
ETDRS-DRSS: Early Treatment Diabetic Retinopathy Study-Diabetic Retinopathy Severity Scale
NPDR: Non-proliferative diabetic retinopathy
PDR: Proliferative diabetic retinopathy
