# Supplementary material for: Progression of diabetic retinopathy in a longitudinal real-world study of patients in primary care
Source: BMC Ophthalmol. 2025 Oct 6;25:547. doi: 10.1186/s12886-025-04307-1 (PMC12502510; doi:10.1186/s12886-025-04307-1)
Supplement: Supplementary file 1 — Supplementary Material 1. [file 12886_2025_4307_MOESM1_ESM.pdf]

**SUPPLEMENTARY TABLES AND FIGURES**

**Supplementary Table 1.** Definitions of outcome measures

| Endpoint | Definition |
|---|---|
| ≥2-step DR worsening | Time from the first DRSS assessment (baseline) until first ≥2-step DR worsening on the ETDRS-DRSS |
| Development of CSME[a] | Time from baseline to the first retinal thickening or hard exudates with associated retinal thickening within 500 μm of the center of the fovea |
| Development of PDR[a] | Time from baseline to the first presence of NVD or NVE or vitreous hemorrhage |

*CSME: clinically significant macular edema, DR: diabetic retinopathy, ETDRS-DRSS: Early Treatment Diabetic Retinopathy Study-DR Severity Scale, NVD or NVE: neovascularization of the disc or elsewhere, PDR: proliferative diabetic retinopathy.*

[a]Analyses include only patients with no PDR or CSME at baseline.

**Supplementary Figure 1.** ≥2-step DR Worsening in Eyes With ETDRS-DRSS 47–53 at Baseline. Data from 403 patients and 515 eyes.

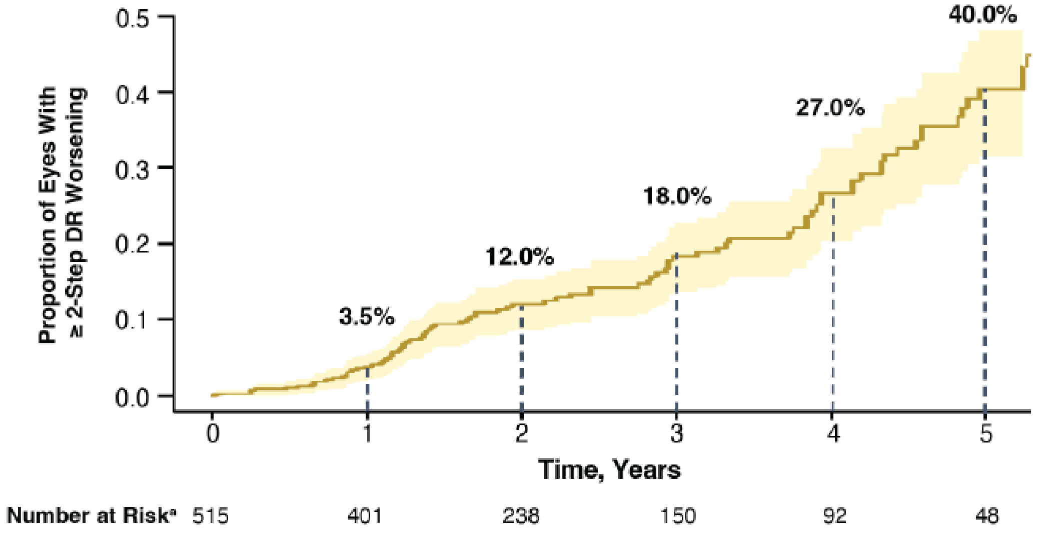


[a]Number of eyes at risk of developing DR at each year. Shaded region indicates 95% CI.

*CI: confidence interval, DR: diabetic retinopathy, ETDRS-DRSS: Early Treatment Diabetic Retinopathy Study-DR Severity Scale.*
